# Methacrylate Polymer Monoliths for Separation Applications

**DOI:** 10.3390/ma9060446

**Published:** 2016-06-03

**Authors:** Robert J. Groarke, Dermot Brabazon

**Affiliations:** 1 Advanced Processing Technology Research Centre, Dublin City University, Collins Avenue, Dublin 9, Ireland; dermot.brabazon@dcu.ie; 2 National Sensor Research Centre, Dublin City University, Glasnevin, Dublin 9, Ireland

**Keywords:** monoliths, methacrylate, porous materials, stationary phase, chromatography, microfluidics, additive manufacturing

## Abstract

This review summarizes the development of methacrylate-based polymer monoliths for separation science applications. An introduction to monoliths is presented, followed by the preparation methods and characteristics specific to methacrylate monoliths. Both traditional chemical based syntheses and emerging additive manufacturing methods are presented along with an analysis of the different types of functional groups, which have been utilized with methacrylate monoliths. The role of methacrylate based porous materials in separation science in industrially important chemical and biological separations are discussed, with particular attention given to the most recent developments and challenges associated with these materials. While these monoliths have been shown to be useful for a wide variety of applications, there is still scope for exerting better control over the porous architectures and chemistries obtained from the different fabrication routes. Conclusions regarding this previous work are drawn and an outlook towards future challenges and potential developments in this vibrant research area are presented. Discussed in particular are the potential of additive manufacturing for the preparation of monolithic structures with pre-defined multi-scale porous morphologies and for the optimization of surface reactive chemistries.

## 1. Introduction

Monolithic materials are increasingly utilized in the separation of both chemical and biological molecules. The term monolith in separation science refers to a single discrete piece of porous material used for the separation or pre-concentration of analytes. This is achieved by passing the analyte within a fluid across a monolith material, which has specific macropores, mesopores and surface chemistry. Pores are defined according to the International Union of Pure and Applied Chemists (IUPAC) in the following way: micropores < 2 nm, mesopores a size range of 2–50 nm, while macropores > 50 nm [1]. Over the years, numerous compounds have been investigated as candidates for the efficient and highly-resolved separation of both chemical and biological species. After this introduction to monolith materials, this paper presents a detailed overview of methacrylate-based polymer monoliths and their applications.

The ability for monoliths to be used as separation media has been known since the 1950s, when porous gels were reported by Synge and Mould [2]. Later, in the 1970s, the forerunners of modern polymer monoliths were fabricated as porous polyurethane foams. Modern monolith materials which were more readily embraced by the chromatography community first appeared in the 1990s due to the seminal work by Švec, Tanaka, and others [3,4]. Such work has been the subject of numerous reviews, and has encompassed a diverse range of materials including, for example carbonaceous monoliths, silicate based materials, inorganic monoliths, and organic polymers, such as vinyl esters, methacrylates, polystyrenes, ethylene glycols, and acrylonitrile-butadiene-styrene (ABS) [5,6,7,8,9,10,11,12,13,14,15,16,17,18,19]. This list does not cover the wide range of functional groups which has been investigated, in order to tailor the monolith stationary phase to a particular analyte or separation mechanism [8,9,10,13,15,20,21,22,23,24]. A critical feature of polymer monoliths is that they possess a large number of smaller pores, which furnishes them with a higher surface area, which is desirable for increased separation efficiencies. Silica monoliths achieve good separation efficiencies by virtue of their high surface area, which can be 10’s–100’s m^2^/g, depending on the preparation conditions. Higher surface areas and greater numbers of smaller pores lead to an increase in potential interaction sites for the analytes in the mobile phase, therefore, in theory, leading to better separation efficiencies. While smaller pores are important for separation, the presence of larger pores is important as these allow for sufficient flow of the mobile phase through the monolith in order to avoid high back pressures, which would inhibit separation and potentially cause structural defects in the monolith, High Performance Liquid Chromatography (HPLC) pump, or problems with tubing connections to the HPLC instrument. Monolith columns can thereby exhibit low flow resistance compared to packed bead columns allowing separations at higher flow rates, which is attractive from the point of view of scaling up separation processes. The monomer units of polymer monoliths can be functionalized prior to the polymerization step or functionalized *in situ* after the polymerization step. Functionalization is discussed in more detail in Section 2.4. Due to these features, polymer monoliths display efficient separations of larger molecules such as proteins.

Of the diverse range of monolith materials, methacrylate polymers have received more attention than others. This can be attributed to their relatively ease of preparation, functionalization [9], and that they can be fabricated with a high degree of macroporosity. The necessity of efficiently obtaining and identifying pure samples of DNA, plasmids, nucleic acids or other biomarkers, as well as pure chemical species, such as pharmaceutical products, has been the driving force behind much of the research into methacrylate monolith development. Separations, amplification, and detection capabilities for these analytes with methacrylate monolithic columns are presented and discussed in this review.

## 2. Methacrylate Polymer Monoliths

**Figure 1:**
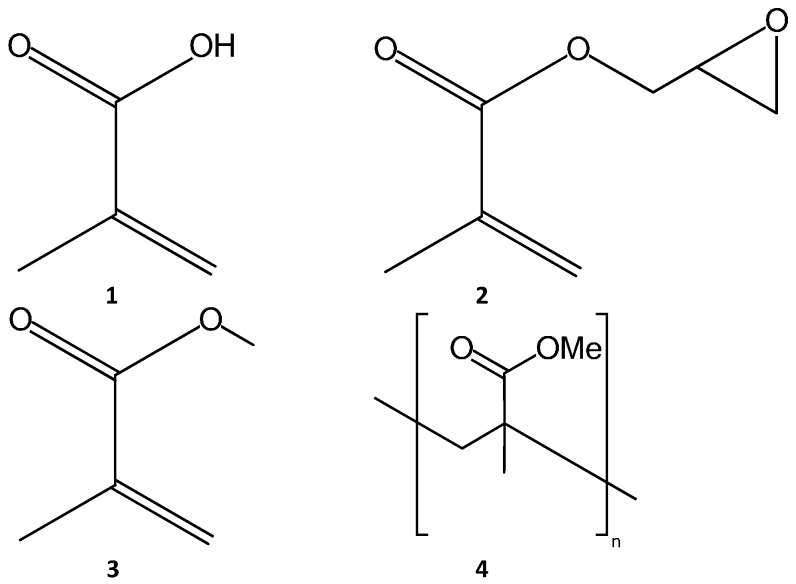
Chemical structures of (**1**) methacrylic acid; (**2**) glycidyl methacrylate; (**3**) methyl methacrylate; and (**4**) PMMA.

Methacrylate is a term for any derivative of methacrylic acid, such as glycidyl methacrylate and methyl methacrylate, which are common monomer precursors, while Poly Methyl Methacrylate (PMMA) is a common monolith material (see Figure 1). As a monolith material, PMMA is presented as a linear structure. Glycidyl methacrylate is a very commonly used monomer as it contains an epoxy group allowing a wide variety of functionalizations [3,15,25,26,27,28,29,30,31] and is also used as a grafting agent [32]. Other methacrylate monomers include methyl methacrylate [33], ethyl methacrylate [34], butyl methacrylate [35,36,37], and octadecyl methacrylate [38]. Methacrylic acid [39,40,41] and hydroxyethyl acrylates [42,43] have also been investigated in this role. The latter are used in the polymer mixture to tailor the hydrophilicity of the resulting stationary phase.

Methacrylates are generally moderately polar due to the presence of carbonyl and ester bonds, however, they are more commonly used in reverse phase chromatography. As a result of the mixed polar/non polar groups and ease of preparation/functionalization, methacrylate monoliths have been prepared for a number of different methods of chromatographic separation, such as ion exchange [44], Hydrophilic Interaction Liquid Chromatography (HILIC) [45], and Reverse Phase [38]. This includes application for the separation of proteins and peptides [27], polymer separations [46,47] as well as alkyl benzenes and other small polar molecules [48,49,50].

### 2.1. Morphology of Monoliths

**Figure 2:**
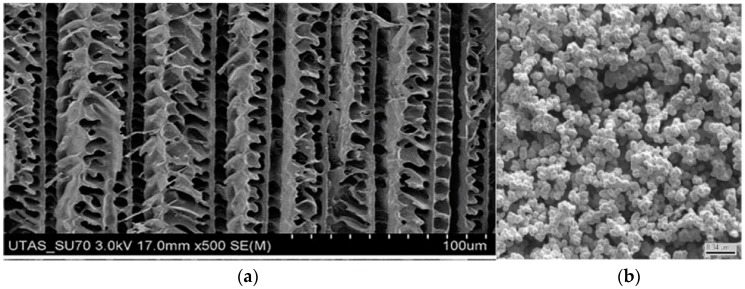
SEM images of (**a**) poly(ethyleneglycol) diacrylate (PEGDA) monolith formed via directional freezing, reproduced from [52] with permission of The Royal Society of Chemistry; and (**b**) a glycidyl methacrylate co- Ethylene DiMethacrylate (EDMA) monolith, reproduced (adapted) with permission from [9], Copyright 2007 WILEY-VCH Verlag GmbH & Co. KGaA, Weinheim, Germany.

The morphology of methacrylate monoliths have been extensively studied using Scanning Electron Microscopy (SEM) as a means of correlating preparation conditions with observed performance of the monolith in its separation and/or pre-concentration capability. Two examples of SEM images of acrylate-based monoliths are poly-ethylene glycol diacrylate and glycidyl methacrylate, as shown in Figure 2. It should be noted that while the structure in Figure 2a appears to be ordered and unidirectional, the separation achieved using these monoliths for proteins and lysozymes was actually quite poor, indicating the complex relationship between structure and performance for these materials. The monolith material in Figure 2b appears to be more isotropic in structure. Other monoliths fabricated using similar synthesis methods showed similar structural patterns and varying levels of porosity, which were dependent on the choice of porogenic solvent used in their manufacture. These previous works highlight the practical challenges, as well as importance of using characterization methods, such as SEM and Transmission Electron Microscopy (TEM) [51], to analyze monolithic structures. Layer-by-layer analysis methods using automated serial Focused Ion Beam (FIB) etching, coupled with SEM, as well as serial-block face SEM and X-ray micro-tomography, provide the means to better relate monolith fabrication parameters to resulting fabricated architecture characteristics, including pore volume and surface area. Details on 3D reconstruction analysis of polymer monolith architectures are discussed further in Section 2.3.

Methacrylate monoliths have also been fabricated within microfluidic devices or channels; for example, in polymer High Internal Phase Emulsion (polyHIPE) separation columns [53] and in monolithic pre-concentration devices [54,55]. The integration of methacrylate and other monolithic materials into microfluidic devices has benefited both analyte separation and pre-concentration in modular microfluidics as the quest for the miniaturization of chromatographic devices continues [41,55,56,57,58,59,60,61,62,63,64,65,66,67,68,69,70].

One other important preparation method involves the co-polymerization of the functional monomer and cross-linker in the presence of a templating agent yielding a Molecular Imprinted Monolith (MIM). This templating agent is chosen based on the intended application (*i.e.*, what the intended analytes will be) as it will need to be a similar size and shape and possess similar chemical functionality. Acrylate based monoliths which are fabricated using this method have received much attention, including several reviews, [71,72]. They are particularly useful for chiral protein separations, facilitated by a chiral templating agent [73]. The area itself is fascinating and quite extensive and, while it will not be discussed further in this contribution, it really merits a review of its own; however, it remains a very interesting research theme and the interested reader is directed to the above-cited reviews for a more detailed treatment.

### 2.2. Methacrylate Monolith Preparation Methods and Compounds

The preparation of macroporous methacrylate based monoliths was first reported in the literature in the 1990s [3,74]. In these methods, the free radical polymerization reaction is carried out in the presence of a precipitant (a solvent with poor solubility for the polymer), which results in a porous polymer network or monolithic structure. There are two main methods of inducing free radical polymerization reactions which have been reported to yield monolithic structures, thermally initiated reactions and photo-initiated polymerization. Equation 1 shows the general reaction scheme.

(1) $$\text{Monomer}+\text{Crosslinking Agent}\frac{\text{Initiator}}{\Delta \mathrm{or}h\nu }\text{Polymer}$$

The general procedure for preparing a polymer monolith involves adding the chosen monomer units, porogen, initiator, cross-linker, and, if applicable, functional groups, into a capillary or column (for example silica, stainless steel, polyimide), which is then sealed at both ends. Then, the tube is either heated or exposed to light of the appropriate wavelength. Naturally, for the photo-initiated polymerization to work, the tube must be transparent to the chosen wavelength. The polymerization is then allowed to proceed until the polymer monolith of appropriate porosity and surface area is obtained. The unreacted starting materials, cross-linker and porogen are then thoroughly flushed from the column or capillary with a suitable solvent.

**Figure 3:**
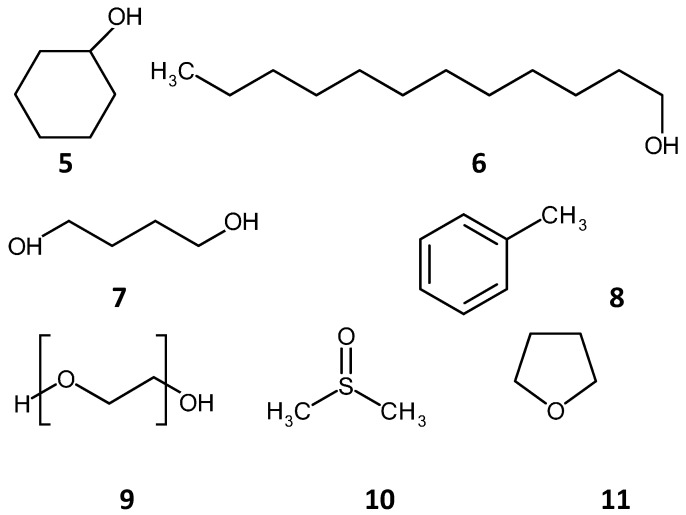
Chemical structures of commonly used porogenic solvents, (**5**) cyclohexanol; (**6**) dodecanol; (**7**) 1,4 butane-diol; (**8**) toluene; (**9**) poly ethylene glycol; (**10**) dimethyl sulfoxide; (**11**) Tetrahydrofuran.

The most commonly used initiator is Azo Bis-Isobutyronitrile (AIBN), generally present in concentrations of 1% (w/w), relative to the monomer and crosslinking agents. Reacting different monomers together in varying ratios can lead to differences in monolithic structure and separation mechanisms. Porogenic solvents cause no chemical change to the monolithic phase and are used in the polymerization reaction to induce pores in the resulting polymer monolith. By varying the amounts of the porogen relative to the monomer content, the surface area and porosity can be controlled. These properties affect the types and sizes of molecules, which can be separated on the polymer monolith. Only a small number of porogenic mixtures are used and are a crucial consideration in the synthetic design of the polymerization. Generally, various mixtures of cyclohexanol (**5**) and dodecanol (**6**), 1,4 butanediol (**7**), dimethyl sulfoxide (**10**) and tetra hydro furan (THF) (**11**), are utilized as the porogenic solvent, see Figure 3 for chemical structures.

Polymerization reaction time is another critical factor in the preparation of methacrylate monoliths, as with other polymer-based materials. Švec noted, early on in the research into methacrylate polymer monoliths, that increasing the polymerization time (from 1 h to 22 h) resulted in a dramatic reduction of the surface area and porosity of the polymer (500 m^2^/g and 3.8 mL/g to 120 m^2^/g and 1.1 mL/g, respectively) [74]. Interestingly, the nature of the thermally initiated polymerization reaction (bulk *vs.* suspension) was also reported, in the same contribution, to have a marked effect on the subsequent porosity without necessarily affecting the surface area or pore volume to the same extent.

Photo-initiated polymerizations of methacrylate monomers were reported later in the 1990s by Viklund *et al.* [75] using UV lamps (365 nm) for 60 min. These yielded monoliths with significantly lower surface areas (11–165 m^2^/g) and macro-pores. They have also, more recently, been performed using LED sources with 600 nm [76] and 660 nm [10] wavelengths.

**Figure 4:**
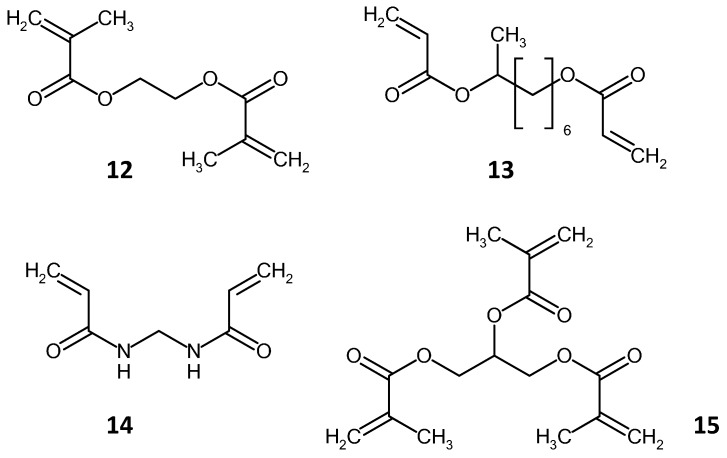
Chemical structures of commonly used cross-linking agents for polymerization reactions. Agents are: (**12**) ethylene glycol dimethacrylate; (**13**) 2-methyl-1, 8-octanediol dimethacrylate; (**14**) methylene bis acrylamide; (**15**) tri methylol propane trimethacrylate.

Some common cross-linker compounds are shown in Figure 4. The cross-linking is via the vinyl bonds present in all of these compounds. Crosslinking agents are responsible for the amount of swelling of the monolith during the polymerization step, and therefore can also affect the void volume of the monolith. The swelling is dependent on the vinyl character of the cross linker, *i.e.*, the mono-vinyl *vs.* di-vinyl content. In this context, mono-vinyl refers to the presence of a single C=C group on the aromatic ring in the crosslinker.

Another methacrylate polymerization reaction, which can be utilized is the living polymerization technique. Living polymerization refers to polymerization reactions where the polymer chains grow at more or less the same speed, thereby giving a polymer mixture of similar chain lengths. This is achieved because in, such reactions, termination and chain reactions do not occur and, therefore, the addition of each new monomer unit results in a re-activation of the chain growth. The reaction exists as an equilibrium state composed of an active and a dormant species, whichever species is favored by the equilibrium determines whether the reaction proceeds or stops. These types of polymerizations (Atom Transfer Radical Polymerization (ATRP) and Reversible Addition Fragmentation Transfer (RAFT)) can be used to graft chemical moieties on to substrates [77,78,79]. The advantage of such techniques is that they produce polymer surfaces that can be further grafted or functionalized. There are a number of methacrylate-based monomers that have been used in polymerization reactions to produce monolithic stationary phases. The most common cross-linking agents in polymer monoliths are ethylene glycol dimethacrylate (**12**, Figure 4) and ethylene glycol diacrylate.

### 2.3. Structural Properties

There are a number of factors that will impact the resulting physical structure of the polymer, reaction temperature, monomer ratios, cross-linker ratios, time of polymerization, composition of porogenic solvent solution, as well as the presence and nature of functional groups on the surface. The subject of functionalization shall be discussed in greater length in the next section. The preparation method utilized is also an important factor. As noted in Section 2.1, the preparation method can affect the resulting structure, such as the effect of increased polymerization time on reducing surface area and porosity. Monolithic structure is routinely investigated using techniques such as SEM, Brunauer-Emmett-Teller (BET) Assay, to obtain surface area and porosity values. More recently, the use of focused ion beam (FIB) sectioning of carbon monoliths has been reported by Vasquez *et al.* [80]. The 3D physical structures of carbon monoliths were measured using a serial FIB and SEM procedure in order to obtain monolith porosity level, pore volume and pore size. These section images (100 images at 100 nm spacing) were compiled into a 3D rendering of the porous structure. The calculated macroporosity of the monolith agreed well with the measured porosity from Mercury Intrusion Porosimetry (MIP). These 3D reconstruction methods should become very useful in future characterizing and understanding of how monolithic structures are generated and how they affect the flow dynamics and mass transport within the monolith. Aggarwal *et al.* used a similar approach for the characterization of PEGDA monoliths, [78], the data gathered from the sectioning and 3D reconstruction of the monolith was used to extrapolate values for tortuosity, porosity and homogeneity of the monoliths. This data could then examined to identify which factors affected chromatographic performance of the monoliths. Three-dimensional SEM analysis of the PEGDA structure showed short range heterogeneity, which should have given a higher interchannel eddy diffusion, and, therefore, a reduction in separation efficiency [78]. However, in such analysis the trans-channel and trans-column dispersion mechanisms at larger dimensional scales also need to be taken into account. In the case of the PEGDA structure, the high macro-scale dispersion is expected to have resulted in a lower overall interchannel eddy diffusion value. This would results in a lower value for the “A” term in the van Deemter equation (Equation (2)), *i.e.*, a lower separation efficiency.

(2) HETP = A + B/u +C × u

where HETP is the Height Equivalent to a Theoretical Plate, A is the Eddy Diffusion Term, B is the Random Diffusion Term and C is the Mobile Phase induced Mass Transfer within the particle, u is linear velocity in ms^−1^.

Recently, the first use of Scanning Transmission X-ray Microscopy (STXM) to analyze methacrylate monolith structures was reported [81]. From the STXM data, it was also possible to distinguish the Butyl Methacrylate (BuMA)-EDMA co-polymer from the di-vinyl benzene nanoparticles. This work represents an interesting characterization tool however it remains a specialized technique at this time.

Porosity and surface area are conventionally measured via a number of techniques including Brunauer-Emmett-Teller (BET) and MIP measurements [80]. A more recent technique applied for monolithic characterization is serial block face SEM. This has been utilized as a promising analytical tool for the analysis of polymer monolith internal structures [82]. This latter work showed two different macropore types and an increasing porosity moving from the outer rim of the column to the middle. This variation was attributed to variations in the temperature profile throughout the capillary during the formation of the polymer. Other changes in physical structure were noted by Laher and co-workers [83] in which they used AFM in the Force-Indentation mode to investigate the extent of crosslinking within globules of commercially available methacrylate monoliths. This work demonstrated a clear difference in the hardness of the globule, with the hardest sections being in the center and progressively lower hardness measured towards the globule edges.

### 2.4. Functionalization Routes and Functional Groups

A large number of functional groups have been investigated and numerous chemical strategies have been developed over the years. The goal has always been to tailor the surface chemistries of polymer monoliths to make them suitable for the separation of a wide range of analytes, both polar and non-polar molecules, chemical and biological species, large and small molecules. Initially, polymer monoliths were not exhibiting acceptable separation efficiencies for smaller molecules, and instead performed better in the separation of larger biomolecules. In contrast, silica monoliths have traditionally shown better suitability for the separation of small molecules. Functional groups on methacrylate monoliths include amine groups [44], gold nanoparticles [25,84], antibodies [61], thiol groups [85] and hydroxyl groups [43,45].

#### 2.4.1. Epoxy Group Transformations

**Figure 5:**
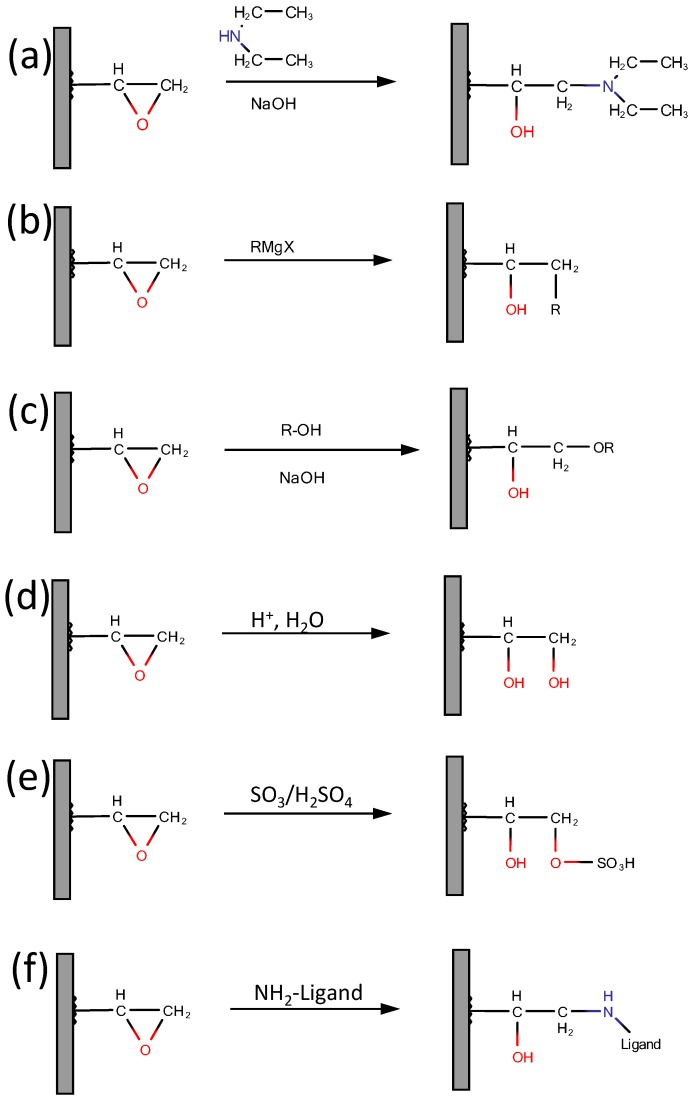
Examples of chemical routes to functional groups on polymer monolith surface using the epoxy group as the starting point, (**a**) functionalization with diethyl amine ethyl; (**b**) Alkyl addition via Grignard reaction; (**c**) alkylation; (**d**) hydrolysis; (**e**) sulfonation; (**f**) biofuctionalization, adapted and redrawn from [9].

As mentioned before, glycidyl methacrylate, (**2**), is possibly the most useful monomer for when functionalization of a monolith is required. This is due to the presence of an epoxy group, which lends itself to numerous modifications, for example amination, alkylation (for C_8_ or C_18_ addition), hydrolysis as well as immobilization of various bio-ligands. The latter is important for a monolith, which is to be used in bio-affinity chromatography. It relies on a reaction between the epoxy or hydroxy groups of the monolith and the NH_2_ group of the ligand. The ligand could be an antibody, protein or peptide. A scheme showing some of the potential reactions using epoxide chemistry is shown in Figure 5. Hydroxyl groups can be added by using hydroxyl-ethyl methacrylate as a co-polymer unit or by acid hydrolysis of existing epoxy groups, (Figure 5, reaction (d)). A detailed discussion of the preparation and modification of methacrylate monoliths by these routes has previously been presented by Vlakh and Tennikova [9] and more recently by Švec and Lv [7].

Anion exchange can be facilitated via the polymerization of diethyl amino ethyl methacrylate and (acryloyloxy) ethyl trimethyl ammonium chloride [44] (which yields a mixture of strong and weak anion exchange groups), and alternatively glycidyl methacrylate and poly ethylene glycol diacrylate can be photo-polymerized and then modified with diethylamino ethyl (DEAE) [86,87]. Similar reactions are illustrated in Figure 5 (reactions (a) and (e)). Hutchinson *et al.* [86] achieved a thirty-fold increase in ion exchange capacity compared with poly styrene-*co*-divinyl benzene monoliths that were functionalized for ion exchange, while Ueki and co-workers [87] reported low flow resistance and reasonable stability of their monoliths over a fourteen day period of use. These examples illustrate the ability to use functionalized monomers in the polymerization step or polymerize standard monomers and then functionalize as desired. The latter method has the advantage of being able to functionalize the surface of the monolith, while the former produces monoliths with functional groups present on the surface and also in the bulk, which may affect the porosity and structural properties of the monolith, particularly if crosslinking or bonding between the functional groups is possible. Methacrylate is somewhat polar, therefore if a methacrylate based monolith is required for a reverse phase separation, treatment of the epoxide groups with an alkyl alcohol will yield the corresponding non-polar alkyl functionality [88] (Figure 5c).

The epoxide groups can also be used to produce a bio-affinity monolith. The production of bio-affinity monolithic stationary phases involves the introduction of a protein, amino acid or peptide moiety to the monolith. Such bio-affinity functional groups can also be introduced via hydroxyl groups on the monolith. A reaction scheme for a modification of a methacrylate monolith with a bio-affinity ligand is shown in Figure 5f, proceeding through the amine group. Cystamine is a common example of a ligand used to modify methacrylate monoliths for bio-affinity and pharmaceutical applications [89].

#### 2.4.2. Nanoparticle Functionalization

**Figure 6:**
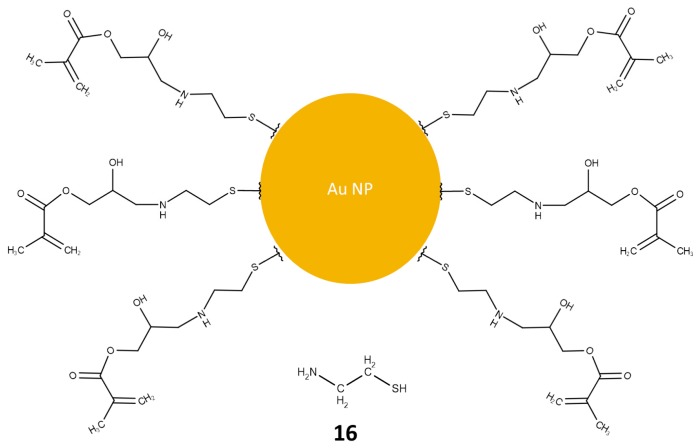
Chemical structure of cysteamine (**16**) crosslinked with glycidyl methacrylate and Au nanoparticle.

Reports of methacrylate monoliths functionalized with nanoparticles have been made by a number of groups. The attention has generally been focused on gold [25,84,85,86,87,88], silver [89,90,91], functionalized gold nanoparticles [92,93], and iron oxide [94,95]. Gold is routinely used due to its well established chemistry [96,97] and silver nanoparticles are also used in Capillary Electrochromatography (CEC) and in Surface Enhanced Raman Spectroscopy (SERS) [98,99]. The abundance of contributions using gold nanoparticles is not unexpected; it lends itself to numerous functionalization reactions, due to its favorable binding to thiol from cysteamine [25,93], amine, and di-sulphide groups. The most commonly utilized source of the gold nanoparticles is HAuCl_4_. The drawback to using cysteamine (Figure 6) is that both the thiol and amine groups can be involved in the functionalization reactions with epoxide groups. The thiol can react to form a less reactive thioether, while cross-linking can occur between the amine groups and the epoxide, reducing the binding of the gold nanoparticles due to steric interactions. Gold has also been used as a means to attach moieties such as cyclodextrins in order to facilitate chiral capillary electro-chromatography [93]. In this case, the thiol groups from cysteamine provided anchoring points for the nanoparticles on the monolith surface.

Silver nanoparticles not been used as much for methacrylate monolithic separations. However, advantage has been made of their good conductivity and electrochemical properties in capillary electrophoresis. Given these excellent properties of silver relative to other functional groups, it is thought that this functionality deserves further investigation. It is potentially the additional reactive nature of gold nanoparticles that has seen the latter gain greater interest. Gold nanoparticles have been used to functionalize methacrylate monoliths for the separation of peptides [25,100], and chiral drugs [93], while silver functionalized monoliths have been used in capillary electrophoresis of sterols, fatty acid methyl esters, tocopherols, and polyaromatic hydrocarbons, as well as in the reverse phase separation of radiolabeled pharmaceutical compounds [89,98].

#### 2.4.3. Click Chemistry

**Figure 7:**
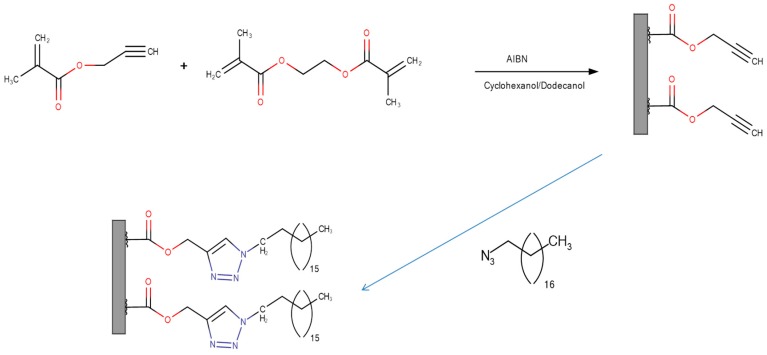
Formation of methacrylate monolith with alkyne functional group, and reaction scheme of the CuAAC reaction for the modification of polymer surface with C_8_ and C_18_ ligands. Redrawn from [111].

Click chemistry, the term coined by Sharpless *et al.* [101] has been heavily utilized by organic chemists for many years as a versatile toolbox of synthetic strategies, many of which have been used in separation science, for the modification of monolithic and other types of separation media [102,103,104,105,106,107,108,109,110]. The mechanistic aspects of the chemistries involved in these reactions are beyond the scope of this article and shall not be considered in detail here. The interested reader is directed to the various reviews cited here for further information [102,103,104,105,106,107,108,109,110]. Briefly, however, one of the biggest advantages of using click chemistry for monolith functionalization is the facile addition of long alkyl chains. Figure 7 gives an example of a reaction scheme reported by Sun *et al.* [111] for the attachment of such groups onto monolith surfaces [102]. Cyclodextrins have also been introduced onto methacrylate monoliths using click chemistry by Guo *et al.* [112,113]. Other reactions such as Michael thiol-ene and thiol-yne additions have been reported [85,107]. This technique is extremely diverse and has great potential in the development of specific, tailored monolithic surfaces with well-defined chemistries.

## 3. Applications of Methacrylate Polymer Monoliths in Separation Science

Methacrylate based monoliths have been investigated in terms of their usage in the separation of chemical and biological molecular species throughout the last 30 years. Many companies now routinely offer monolith based separation columns. One of the best-known commercialized methacrylate based system is the Convective Interaction Media (CIM™, BIA Separations, Ljubljana, Slovenia). In this section, published work on monolithic stationary phases for the separation of chemical and biological analytes are discussed. This review is arranged into two main sections, chemical separations and biological separations. The chemical separation section is further sub-divided into two sections, (1) pre-concentration and solid phase extraction (SPE); and (2) chemical chromatographic separations. The biological separations section is also sub-divided into two sections as follows: (1) amplification of DNA, plasmids, viruses and peptides; and (2) chromatographic separation of biological species, such as proteins, DNA, plasmids and amino acids. This review is focused on methacrylate-based monolithic systems, however, in the case of SPE, some carbon monolith reports are also cited as a base reference, given carbon’s extensive usage as a sorbent material [114,115,116,117,118,119]. Challenges and drawbacks of methacrylate based monolithic media are discussed in Section 3.3.

### 3.1. Separation and Pre-Concentration of Chemical Species

Efficient chemical separations are necessary in order to purify or extract chemicals and analytes of interest from complex environmental or pharmaceutical matrices. Such analytes may include alkyl-benzenes, poly-cyclic aromatic hydrocarbons (PAHs), and chiral pharmaceutical compounds. Pure, unadulterated samples of pharmaceutical compounds are required by law; therefore, the need for efficient chromatographic systems is clear in this regard. However, given the commercial sensitivity in such matters, information about many separation methods is proprietary and not available within the literature.

As previously discussed, a common issue with polymer monoliths in reverse phase mode is that of poor column efficiency for small molecules, and therefore, generally speaking, polymer monoliths have been more often utilized as separation media for larger biomolecules. This trend is also evident from examination of number of published papers for the different applications of methacrylate monolith however the literature also presents small molecule chemical separations. Increasingly though, methacrylate based monoliths have found a role in environmental applications. Environmental pollution is an increasingly important concern in the developed world and therefore, methods of identifying and quantifying the levels of pollution are essential and are actively researched. Purification, pre-concentration, and detection are therefore critical research areas in chromatography. Methacrylate monoliths have played a significant role in this research, as described in the next section.

#### 3.1.1. Solid Phase Extraction (SPE) and Pre-Concentration of Chemical Species

This section discusses the use of methacrylate monolithic stationary phases as materials for the concentration and extraction of chemical species from a solution. Common species investigated using this type of SPE material include; pharma compounds [120,121], PAHs [122,123], chloro-phenols [122], dyes [41], herbicides [124], metal ions [54,125] and basic compounds such as caffeine [126]. The implementation of methacrylate monoliths in microfluidic chips for on-chip SPE has also been investigated [127].

A number of Non-Steroidal Anti-Inflammatory Drugs (NSAIDs) were extracted using a monolith composed of Alkyl-MethAcrylate-ester (AMA), Di-Vinyl Benzene (DVB) and Vinyl-Benzyl Tri-methyl-Ammonium chloride (VBTA) [121]. The composition of the monoliths component (AMA) was varied and the effect of such variations on the extraction performance was investigated. In addition, the ratio of AMA to DVB was varied. It was found that shorter carbon chains on the AMA lead to better extraction for some of the NSAIDs, butyl methacrylate showing particularly good extraction efficiency and reasonable levels of re-usability, exhibiting recoveries ranging between 80% and 95% over 50 uses.

**Figure 8:**
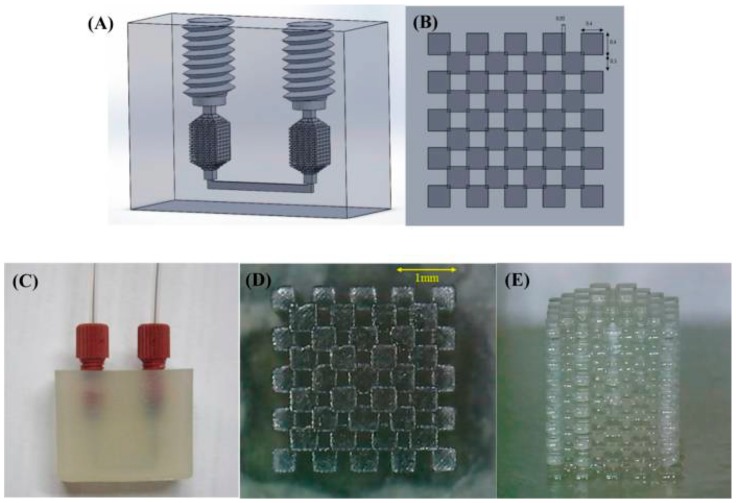
(**A**,**B**) Computer Aided Design (CAD) drawings of the (**A**) pre-concentrator and (**B**) a layer of ordered cuboids in the extraction channel; (**C**) Photograph of the printed device; two flat-bottom female connectors with a piece of Poly Tetra Fluoro-Ethylene (PTFE) tubing were fitted to allow connection to an Flow Injection Analysis (FIA) interface; (**D**,**E**) Photographs of the configuration of the ordered cuboids printed without the surrounding of the extraction channel. Reproduced with permission from [54], Copyright (2015) American Chemical Society.

The application of methacrylate monoliths to the SPE of metal ions and herbicides would be of particular interest to environmental chemists. Three recent reports [54,124,125] show novel methods of applying acrylate monoliths in SPE. Su *et al.* [54] utilized the increasingly popular additive manufacturing method of 3D Printing to fabricate a polyacrylate monolith within a microfluidic chip and demonstrated the effective extraction and pre-concentration of metal ions, such as Mn, Ni, Cu, Zn, Cd and Pb, from sea-water samples. The device was stable at flow rates up to 0.5 mL/min. Good extraction efficiencies were observed and the authors noted the potential of such a fabrication technique to produce SPE materials quickly and with well-defined morphologies, and the integration of such extraction phases within modular multi-component chips. The polyacrylate cube fabricated by the group is shown in Figure 8. This device can also be altered to give monoliths of varying lengths, a factor that can play a role in extraction efficiency. In addition, such commercial polymer resins are easier to prepare, with known curing times.

**Figure 9:**
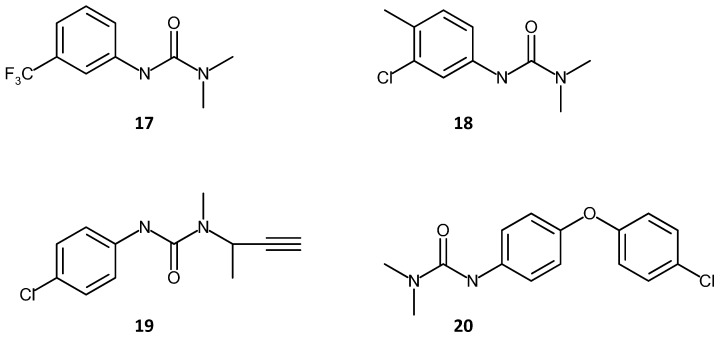
Chemical structures of herbicides ((**17**) fluometuron; (**18**) chlortoluron; (**19**) buturon and (**20**) chloroxuron), used by Lin *et al.* [124].

Lin *et al.* [124] used a 1,6-hexanediol ethoxylate diacrylate (HEDA) monolith for the extraction of phenyl-urea herbicides from water samples. Extraction recovery values of >91% were found for the herbicides. Critically, the detection limits of such compounds were lower than the legal requirement (0.1 ng/mL), showing potential of the system for real world applications. The chemical structures of these herbicides (fluometuron, chlortoluron, buturon and chloroxuron) are shown in Figure 9.

#### 3.1.2. Chemical Separations

Alkyl-benzenes are one of the widely used sets of compounds for testing and evaluating the performance of various chromatographic stationary phases. Their use as test compounds has also extended to monolithic stationary phases. Being non-polar, they are ideal candidates for the evaluation of reverse phase separation performance. The longer the alkyl chain, the more non-polar the molecule, and, hence, the longer its retention time on a non-polar column or monolith. Indeed, one of the first reports on the use of methacrylate monoliths in chromatography discussed the separation of a series of benzene and polystyrene analogues [3]. Carrasco-Correa *et al.* [31] prepared a monolith modified with magnetic iron oxide nanoparticles and separated pesticides and an alkyl-benzene mixture on it.

A significant increase in the retention times of the pesticides and the alkyl-benzenes was observed when the monoliths were functionalized with the nanoparticles. This was attributed to the increased surface area due to the presence of the nanoparticles, and therefore an increase in the interactions between the analytes and the stationary phase. This led to better separation and more resolved peaks, indicating the potential of nanoparticle functionalized monolithic stationary phases. However, the monoliths exhibited no increase in selectivity for the pesticides after modification with the nanoparticles. This was attributed to the possibility that the nanoparticles were themselves covered with a layer of the methacrylate co-polymer. The authors observed that since the nanoparticles had vinyl groups, it was likely that these reacted with not just the methacrylate polymer on the walls of the capillary but that polymer layers appear to have built up on the nanoparticles themselves. Hence, while nanoparticles afford a higher surface area, the lack of improved selectivity for certain analytes can occur due to this unwanted reaction.

Rapid, reproducible separations of alkyl-benzene mixtures were demonstrated by Nesterenko *et al.* [128] using a butyl methacrylate–ethylene di-methacrylate monolith. The same monolith was also used to separate pesticide mixtures. Critically, the monoliths reported in this contribution were produced on a large scale and showed good isocratic separation even for the test solution of small molecules.

Polycyclic aromatic hydrocarbons have also been separated via reverse phase and capillary electro-chromatography on methacrylate monoliths. Ladner and co-workers [68] carried out the polymerization of glycidyl methacrylate and EDMA inside cyclic olefin co-polymer microchannels and used this monolithic stationary phase to separate PAH mixtures via Capillary Electro-Chromatography (CEC). Increased concentration of photo-initiator led to improved separation however the devices suffered from weak anchoring of the monoliths to the Cyclic Olefin Co-polymer COC walls, leading to voids and gaps between the stationary phase and the walls which in turn lead to band broadening. In addition, it was noted that low pressures were required to avoid removal of the monolith. However, it did demonstrate the feasibility of using a single photo-initiator for both the anchoring of the monolith to the capillary walls and the polymerization step.

**Figure 10:**
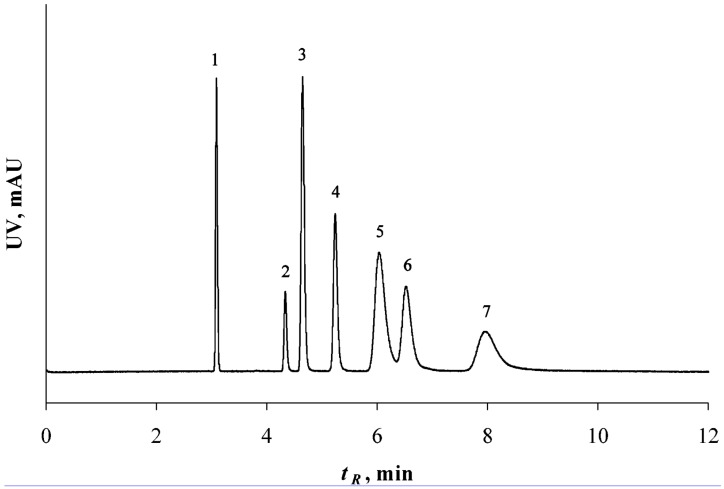
Chromatogram showing separation of a mixture of PAH compounds, on low density methacrylate monolith. Key: (1) thiourea; (2) naphthalene; (3) fluorine; (4) anthracene; (5) pyrene; (6) benz(a)anthracene; and (7) benzo(a)pyrene. Reprinted with permission from [35], Copyright (2005) American Chemical Society.

Eeltink *et al.* [35] prepared a methacrylate ester monolith with a tight pore size distribution and used a PAH mixture to evaluate the performance. Baseline separation of the mixture was achieved via CEC separation, however the authors reported similar efficiencies when the monolith was used in the HPLC mode. Better flow characteristics were obtained using CEC. Interestingly, even though the surface areas of the high-density monoliths in CEC were low, similar retention factors were observed for the separation as on a packed silica column. Figure 10 shows a separation of a PAH mixture on a low-density monolith operating in the CEC mode.

A hexyl acrylate monolith was synthesized via photo-polymerization by Augustin *et al.* [122] and used in the pre-concentration and separation, via CEC, of nine PAH compounds. Good separation of the mixture was achieved, while pre-concentration factors of up to 100 were reported. Interestingly, the monolith was also prepared in a glass microfluidic channel, and separation was achieved in less than four minutes, with the number of plates estimated to be around 200,000/m and concentration factors of around 200 were calculated.

Methacrylate monoliths have also been used for the separation of racemic pharmaceutical compounds. The pharmaceutical industry is risk and change averse, so it would be slow to adopt a new technology, especially one which might be deemed to be evaluating/improving product quality. However, a recent report by Ghanem *et al.* [129] illustrated the effective separation of a number of chiral pharmaceutical compounds including alprenolol, bufuralol, carbuterol, cizolertine, desmethylcizolertine, eticlopride, ifosfamide, 1-indanol, propranolol, tebuconazole, tertatolol and *o*-methoxy mandelic acid. The separation was carried out under reverse phase conditions. Twelve distinct classes of compounds were investigated, as well a number of other compounds listed as miscellaneous, for example, the mandelic acid based compounds. The monoliths contained a cyclodextrin functional group, which facilitated the chiral separations, it was also noted that the separations used more environmentally friendly solvents, and that the fabrication of the polymer monolith was easier and quicker than the fabrication of the silica monoliths also reported in the same contribution. Yu and co-workers [130] prepared a poly (6-azido hexanoic acid (AHA)-*co*-propargyl methacrylate-*co*-ethylene dimethacrylate) monolith via the CuAAC click reaction (as discussed above), for the separation of alkyl-benzenes, phenols, anilines and PAHs. Good separations were obtained for all of these compounds however the plate number for the PAH separation was relatively low (21,000/m–28,000/m). 

Other examples of chemical analytes separated using methacrylate monoliths are phenols, xylene and bi-phenyl [131], anilines (using ion exchange) [132], metal ions (Mn, Co, Cd, Zn and Cu) [32], benzoic acid derivatives [133], as well as capillary liquid chromatography of aflatoxins [50], acids and bases [134]. The extraction of metal ions [32] is one of great importance in environmental chemistry, however it was noted by the authors in this contribution that copper could not be isocratically eluted from the monolith in any reasonable length of time. However, the monolith appears to be suitable for the separation of other divalent metal ions.

### 3.2. Biological Separations and DNA Purification

Biomolecules that have been separated with methacrylate monoliths include genetic material (DNA, RNA, viral plasmids) as well as amino acids, proteins and peptides. Albumin mixtures are commonly separated or extracted using methacrylate monoliths as the stationary phase and these mixtures are often composed of Human Serum Albumin (HSA), conAlbumin (conA), Bovine Serum Albumin (BSA), and Ovalbumin [44,53,135,136,137,138]. Since these albumins are from different sources, and would not normally be found *in vivo* together, they are not necessarily a good example of a typical, naturally occurring protein mixture. However, their separation can be used to give an indication of the performance of the chromatographic system under investigation.

Abnormal levels of serum albumins in the blood can indicate or lead to various medical conditions and therefore it is important to understand the interaction of albumin proteins with drug molecules and other biomolecules present in blood plasma in terms of binding mechanisms and kinetics, and how abnormal levels of albumins lead to the onset of disease. For this, it is necessary to be able to test for albumins and to be able to prepare purified samples from blood plasma matrices in order to measure their concentration successfully. As with some chemical species, albumins and other biomolecules are often present in complex matrices and at low concentrations, which requires pre-concentration of the sample via SPE.

Plasmids are small circular, double stranded DNA molecules that are typically found in bacterial cells. They are separate from the bacterial cells own chromosomal genetic material and can replicate independently from the bacterial cell. Plasmids are known to impart some additional functions to bacterial cells, for example resistance to antibiotics or sterilization treatments. In addition they are also used by biopharmaceutical companies as vectors to induce bacterial cells to produce a drug or other molecule, which the cell would not normally produce due to absence in its own genetic code of the ability to synthesize the molecule. Understanding plasmids is of critical importance for the biopharma industry and the development of new antibiotics in particular, as bacterial strains are becoming increasingly resistant to current antibiotic therapies, even some which have been extremely effective in the past [139].

The separation and purification of peptides is extremely important as peptides can be used to model the interactions of drugs with proteins or antibodies and therefore guide drug development. Antimicrobial peptides are becoming an attractive alternative to antibiotics and are produced by bacteria under carefully controlled culture conditions, however the amounts secreted are small and must also be purified and concentrated. Proteomics research also requires pure samples of peptides and proteins. The analysis of these samples can lead to the identification of disease biomarkers, which can then lead to targeted therapeutics.

The analysis of DNA and RNA yields information about mutations in the genetic code of a patient, which may be identifiable biomarkers for diseases such as cancer. Investigations into the genetic structure of micro-organisms are also extremely important in order to develop strategies to eradicate the pathogenic strains and, as with plasmids, it is important to understand their interactions with each other and with biomolecules and pharmaceutical therapies.

Polymer monoliths have always been considered more suited to the separation of large or biomolecular species, an assumption which is being increasingly challenged by various groups in recent years, as seen in the previous sections of this article. Nevertheless, the bulk of reports involving methacrylate monolithic stationary phases have investigated the separation and purification of biological species such as amino acids, nucleic acids, proteins, DNA, plasmids and viruses. Other less frequently reported compounds have included angiotensin receptor antagonists and microcystins [135]. Various chromatographic modes have been used, such ion exchange, HILIC, Hydrophobic Interaction Chromatography HIC, reverse phase, size exclusion, enrichment and pre-concentration, and bio-affinity, using capillary columns and also microfluidic platforms.

One of the earliest reports of the use of methacrylate monoliths for the separation of biomolecules can be traced to a 1992 contribution by Švec and Fréchet [3], which used continuous polymer rods modified with diol and amine moieties to separate protein mixtures via ion exchange. They suggested that the work may improve the potential of such polymer rods for use as stationary phases in the future. Since that contribution, a large body of work including reports, reviews and books, by many groups has been produced discussing the separation of proteins and other biomolecules using methacrylate stationary phases, in both ion exchange [44,130,136,138] and reverse phase modes [85,130]. This section will be divided into subsections dealing first with the separation of proteins, peptides and amino acids, then discussing DNA separation and enrichment, including the separation of plasmids, viruses and other biomolecules. A more recent contribution discussed the role of CIM™ monolithic supports for the separation of large biomolecules [140].

Section 3.2.1 discusses the amplification and pre-concentration of DNA, plasmids viruses and peptides using methacrylate monoliths as the support material and Section 3.2.2 presents the application of methacrylate monoliths in the chromatographic separation of proteins, peptides and amino acids, involving reverse phase, size exclusion, immuno-affinity and HIC.

#### 3.2.1. Amplification and Pre-Concentration of DNA, Plasmids, Viruses and Peptides

In this section the use of methacrylate monoliths for the enrichment, purification and separation of DNA and peptides are presented. The DNA is generally in the form of plasmid or genomic DNA in these applications. The separation and purification of plasmids has significant usage within the pharmaceutical industry (in the production of plasmid vaccines and plasmid based therapies) and therefore on the improvement of patient health. Consequently, there has been a significant amount of investigation into the purification of plasmid DNA carried out by many research groups, as discussed below [141,142,143,144,145]. Some Good Manufacturing Practice (GMP) validated methods have also been reported [140,146]. A comprehensive review of the extraction and purification applications of polymer monoliths with an emphasis on biomolecular species was published by Jungbauer and Hahn [147] and the reader is directed to this article and the references contained therein for further detailed information on this area, as well as a more recent review published in 2012 [148]. The plasmid nucleotides are negatively charged; therefore, it follows that anion exchange chromatography has been a popular technique in the enrichment of plasmid DNA. As discussed previously, DEAE and amine groups can be used as effective anion exchange functionalities on methacrylate monoliths. Plasmids are generally much larger and exhibit much lower diffusion efficiencies than proteins do. Therefore, they bind to more sites than proteins, meaning lower binding efficiencies for polymer monoliths. Initially such amplification work was focused on small (<10 kb) plasmid samples [141]. More recently, the process has been seen to be scalable and reproducible [142],]. In this latter work, of the cell genetic material 99% of the genomic DNA, proteins and RNA were removed from the mixture, leaving the highly purified plasmid DNA behind [149]. This was achieved using Hydrophobic Interaction Chromatography (HIC) and anion exchange functionalities on the same monolith, indicating the usefulness of multi-modal chromatography (MMC) for this application.

Larger plasmids have also been investigated as in the case of a 62 kb plasmid, which was purified using a commercial CIM™ monolith with DEAE functional groups [150]. Enrichment factors of ~100 were obtained, and the authors noted that the column loading was much lower than the maximum possible loading, indicating scope for further improvement of the purification factor.

The effect of plasmid size on the binding efficiency of the monolith was studied by Bicho *et al.* [151] using a glycidyl methacrylate-*co*-ethylene di-methacrylate monoliths which in some cases were grafted with carbonyl di-imidazole functional groups. A number of different plasmid sizes were investigated in this work, of around 2, 6, 10 and 14 kbps. All of these plasmids were resistance or R plasmids, conferring antibiotic resistance to a bacterial cell should they be present in it. They found that all the plasmids could be separated using the same procedure, and that the flow rates used in the study did not have a strong effect on the dynamic binding capacity, a feature previously noted for polymer monoliths. The monoliths were seen to have lower binding capacities for the larger plasmids. In addition, pH was seen to be an important factor, at lower pH, interactions between the phosphate groups in the plasmid DNA and the carbonyl di-imidazole groups. Critically, the structure of the plasmid DNA was not affected by the separation procedure, at lower pH values.

Urthaler *et al.* also investigated the purification of small plasmids with the intention of possible scaling up of the process. [146]. The DEAE modified CIM™ monoliths showed better binding capacities, larger pore sizes and better recovery of the pDNA. The 800 mL monolith exhibited 2000 mL/min flow rates under nominal back pressures and showed high throughput for DNA purification. Scale up to eight liters monolith volume is expected to be possible however the authors noted that flow rate would need to be reduced in order to avoid prohibitively high back pressures due to the increased column length required. It was observed that the elution profiles did not change with varying flow rates over the range of 800–1600 cm/h. More recently, Shin *et al.* [143] used a CIM™ monolith treated with a CuCl_2_ solution to purify plasmid DNA from other species in the matrix such as endotoxins, and then purified the DNA further using another CIM™ monolith phase. The monolith mediated separation and purification yielded a threefold increase in production of the plasmid compared with a packed column stationary phase. Other groups have also used similar monolithic stationary phases for plasmid separations [144,149], and bacteriophage separation [145].

The removal and purification of viruses are important and complicated process steps in environmental samples and pharmaceutical processes. Rački *et al.* [152] reported the removal of viruses from a wastewater sample using a methacrylate monolith functionalized with quaternary amine moieties. Purification of the water samples from the 5 enteric viruses tested was achieved to a level where the viruses were not detected using Real Time-quantitative Polymerase Chain Reaction (RT-qPCR). Baculoviruses are an important source of recombinant drugs and biopharmaceuticals and an efficient purification process for this precursor was demonstrated using CIM™ monoliths modified for anion exchange [153]. Both strong and weak anion exchange monoliths showed good binding characteristics, with the quaternary amine (strong anion exchange) exhibiting up to three times more binding efficiency. Good recovery of 33%–70% was observed for the virus under gradient elution, increasing the HEPES (4-(2-Hydroxyethyl) piperazine-1-ethanesulfonic acid) buffer content led to increased recovery. These % recovery values increased further to almost 90% in some cases, with the addition of a CIM™ epoxy monolith to remove lipid content in the sample matrices. Viral enrichment factors of around 51 can be achieved in a single purification cycle.

#### 3.2.2. Chromatographic Separation of Proteins, Peptides and Amino Acids

Krajnc *et al.* [53] fabricated a polyHIPE type methacrylate monolith to separate a mixture of proteins (myoglobin, conA and soybean trypsin inhibitor) and found that this type of monolith yielded separations under the applied conditions and also showed a similar performance to the commercial CIM™ monolith, but showed a slightly higher dispersion and lower surface loading which may explain the poor resolution and broad peaks, particularly for the conalbumin and soybean trypsin analyte peaks. However the authors noted that neither the polymerization reaction nor the modification of the monolith with DEAE was fully optimized.

Jandera *et al.* [154] used a methacrylate column and inter-particle methacrylate monoliths to separate a protein mixture composed of insulin, trypsin, BSA and lactoferrin, and compared the performance of the prepared monoliths for this mixture, against the commercial Chromolith™ Silica-C_18_ columns which are often used as the standard monolithic media for performance comparisons. The prepared monoliths showed better separations than the commercial ones and exhibited shorter retention times. A number of different monolith columns were used, and the hybrid inter-particle monoliths showed improved performance compared with the “whole-volume” monoliths, yet in general, most exhibited relatively poor performance by modern standards.

**Figure 11:**
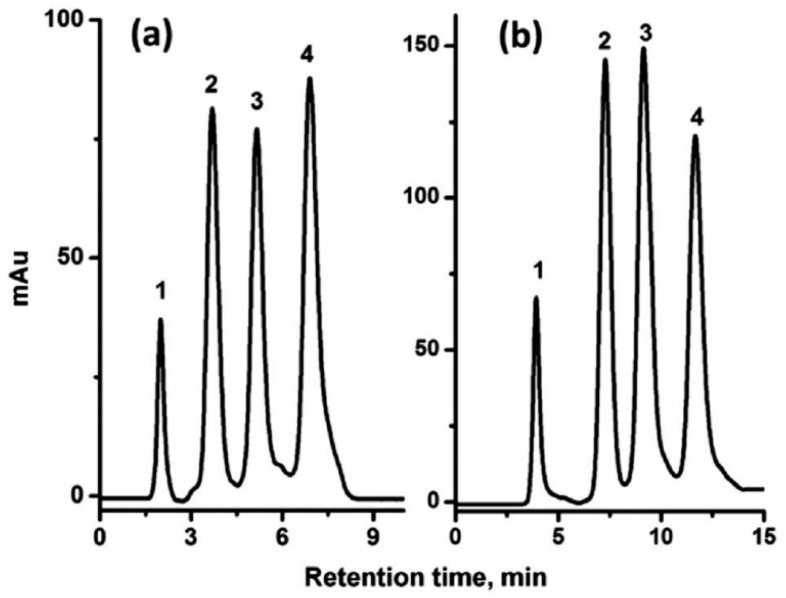
Reverse Phase separation of protein mixture on two different Lauryl methacrylate monoliths, (**a**) fabricated using photo-initiated polymerization and (**b**) fabricated via thermally initiated polymerization. Peaks are: (1) impurity; (2) ribonuclease A; (3) Cytochrome C; and (4) Myglobin. Reproduced from [85] with permission from The Royal Society of Chemistry.

However, a markedly different picture is reported some six years later using a more conventional monolith. Lv and co-workers [85] prepared lauryl methacrylate based monoliths modified with either C_12_ or sulfonate groups. These were then used to facilitate the reverse phase separation of a mixture of proteins, as shown in Figure 11. The resolution achieved was reasonable, almost baseline however difficulties with separating smaller molecules were noted.

Chen *et al.* examined the effect of polymerization time on the retention times and separation efficiencies of methacrylate polymers modified for cation exchange and found that increasing the polymerization time led to longer retention times but that the back pressures did not vary appreciably [138]. However, the morphologies of the monoliths did vary quite dramatically which would be expected to contribute to altered separation characteristics.

Anion exchange mediated separation was reported by Li *et al.* [44]. Strong and weak anion exchange separations of acidic proteins were achieved, with reasonable resolution, in times of 20–30 min. Amine functional groups were used to affect the anion exchange. Cation exchange via vinyl sulfonic acid moieties on a poly ethylene glycol di-acrylate monolith were reported by Gu and co-workers [136]. Strong cation exchange was observed, with near—baseline resolution and separation times of 30–40 min reported. It was noted by the authors that tuning of the level of hydrophobicity in the polymer is crucial in order to minimize non-specific interactions. The linker molecule and the backbone polymer chain are both important factors in this regard. Proteins have also been separated using immuno-affinity [61], HIC [43], and Size Exclusion Chromatography (SEC) [155]. Most recently, Yu *et al.* [130] used methacrylate columns as stationary phases for Normal phase, reversed phase, cation exchange, HIC, and HILIC type chromatographic separations for a number of different types of analytes, including proteins. A protein mixture of BSA, Ovalbumin, Myoglobin, Lysozyme and Cytochrome C were well separated within 40 min, albeit using an acetonitrile/trifluoroacetic acid based linear gradient elution.

**Figure 12:**
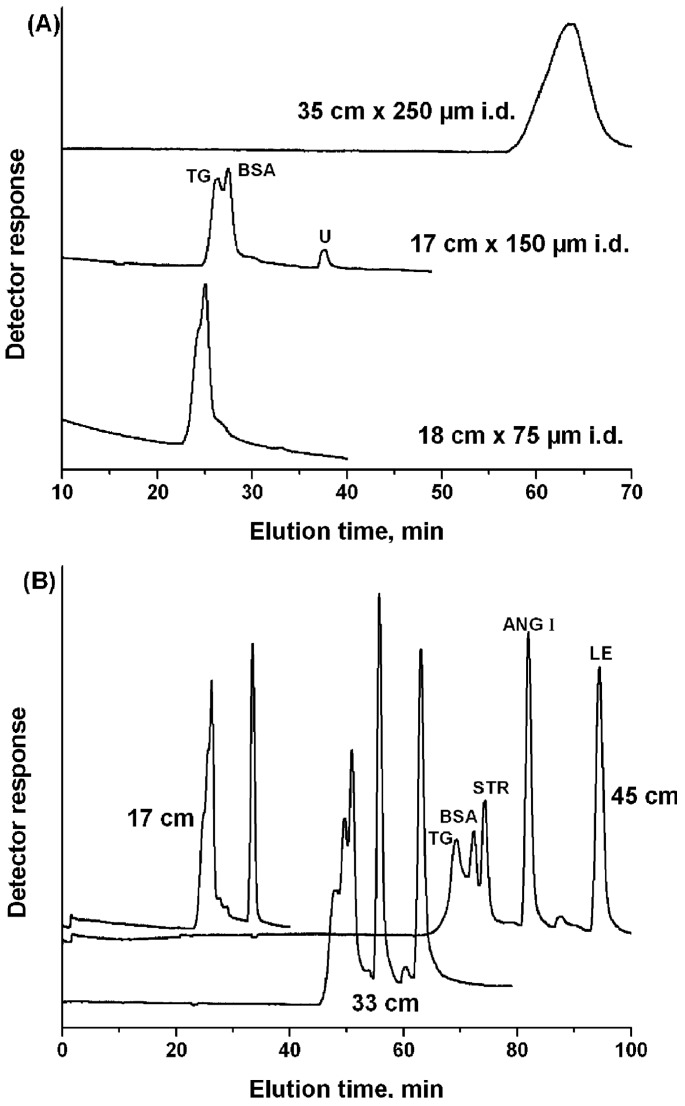
SEC separation on methyl ether acrylate monolith using different column lengths (**A**) and diameter (**B**). Mobile phase was 20 mM phosphate with NaCl. Key: TG: Thyroglobulin; BSA: Albumin; STR: Trypsin Inhibitor; ANG1: Angiotensin 1; LE; Leucine encephalin. Reprinted with permission from [155], Copyright (2009), American Chemical Society.

Li *et al.* [155] investigated the effect of column length and diameter of a Poly Ethylene glycol-Methyl Ether Acrylate based monolith on the size exclusion based separation of protein mixtures. They found that increasing column length improved separation and resolution, but also increased the separation time. Some data from the report is shown in Figure 12. However, the effect of internal diameter appears inconclusive. More systematic data is required in order to fully explain the effect or lack thereof, of the column diameter, whether this is a one off result or something altogether more interesting. If column diameter does not play an obvious role in such separations at these diameters then what is the range of values at which this continues to be the case? A design of experiments where the length is varied with diameter kept constant and vice-versa would have been a useful exercise in order to better understand this result. The monolith was reported to exhibit better separations than a packed column of the same diameter, the interesting point to note is that the monolith was significantly shorter than the column (17 cm *vs.* 57 cm).

He *et al.* [70] investigated the separation of protein mixtures via electrophoresis, using hydrophilic and hydrophobic monomers (hydroxyl-ethyl methacrylate and butyl methacrylate respectively), in the polymer mixture. Baseline separations were reported, using the hydrophilic monomer-containing monolith. Yeast [156] and, more recently, whey [157] proteins have also been separated. In the case of the yeast protein mixture, a multi-dimensional separation setup was employed, involving strong cation exchange, followed by reverse phase chromatography. The separation took a relatively long time (12 h) to complete.

Amino acids have also been investigated on methacrylate monoliths. Two particular examples are discussed here. The first is the separation of a series of dansylated amino acids on an ethyl methacrylate based monolith which was cured using electron beam (e-beam) curing [34]. Electron beam curing involved a linear accelerator, and irradiation with electrons to a dose of 22 kGy (kiloGrays) was performed which initiated the formation of the free radicals necessary for the polymerization step. Again, the comparison was made with commercial monoliths by using Chromolith™ (Silica-C_18_, Merck, Darmstadt, Germany) as the standard. The e-beam curing method was found to be an easy procedure, and amenable to large diameter monoliths. It would be interesting to investigate the uniformity of the porosity of monoliths fabricated using this method, and in particular to see if they exhibit the same variation at the wall as observed in methacrylate monoliths produced from thermally induced polymerizations.

**Figure 13:**
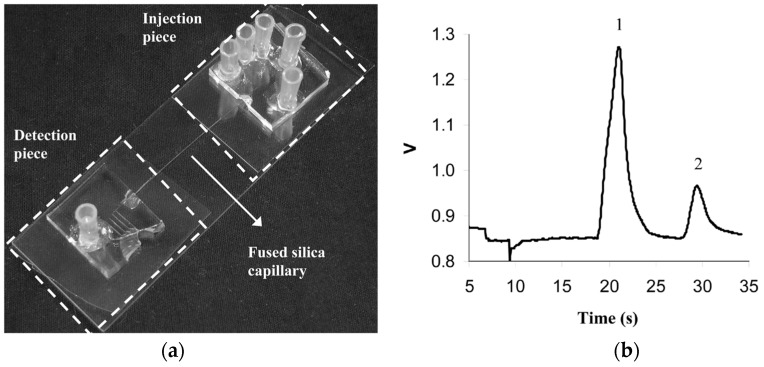
(**a**) PDMS Chip with Butyl Acrylate Monolith in a Silica Capillary; (**b**) Separation of Arginine-NDA (1) and Dopamine-NDA (2) on Acrylate Monolith on PDMS Reproduced with permission from [158], Copyright 2007 WILEY-VCH VerlagGmbH & Co. KGaA, Weinheim, Germany.

**Figure 14:**
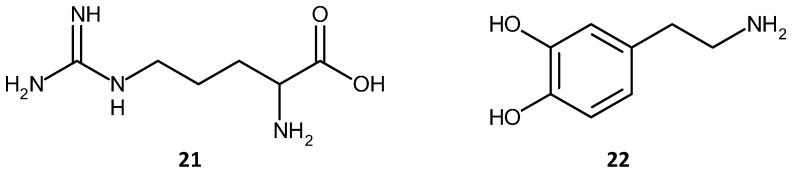
Chemical structures of Arginine (**21**) and Dopamine (**22**).

The second example of amino acid separation is a separation carried out on a microfluidic device. In recent years, the integration of separation and sensor media into microfluidic devices has garnered interest. The reasons for the increased interest are the lower analyte and solvent volumes required, the potential for autonomous, remote sensing, testing at point of analysis, instead of having to remove the sample to the lab, and the development of rapid, cheap, and disposable devices. In this second example [158] an acrylate monolith was fabricated in a silica capillary which was then integrated into a Poly Di-Methyl Siloxane (PDMS)/Glass chip. The schematic of the microfluidic chip for this separation and a chromatogram reported by the group are shown in Figure 13. The separation of Arginine—NDA, (NDA: Napthalene 2,3-dicarboxyl-aldehyde) and Dopamine-NDA, (**21**) and (**22**), shown in Figure 14, was performed using electro-chromatography.

**Figure 15:**
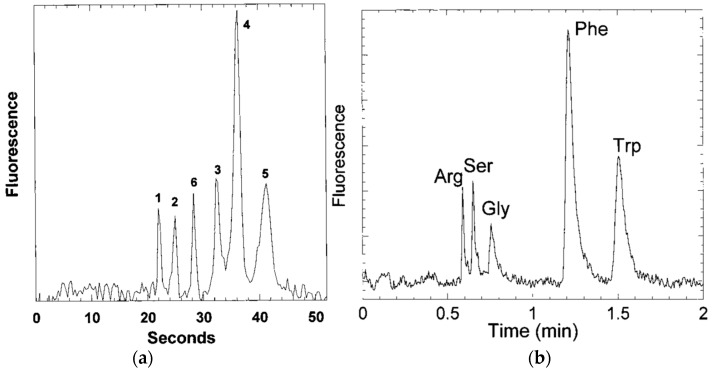
(**a**) Separation of peptide mixture on acrylate monolith. Peptides are (1) papain inhibitor; (2) proctolin; (3) Opioid peptide (R-casein fragment 90–95); (4) Ileangiotensin III; (5) angiotensin III; and (6) GGG; (**b**) Separation of amino acid mixture on same monolith. Image adapted with permission from [159]. Copyright (2002), American Chemical Society.

A glass chip was also used for amino acid separations using an acrylate based monolith [159]. The chip/monolith system achieved reasonable separation of a mixture of five amino acids (Arg, Ser, Gly, Phe, and Trp), see Figure 15. Critically, it was found that the chip could be re-used, indicating a potential for commercialization of the system.

### 3.3. Challenges for the Development of Methacrylate Monoliths

#### 3.3.1. Poor Small Analyte Separation Efficiencies

Methacrylate-based monoliths do have limitations that need to be addressed. These form the basis for both current and future research focus. Perhaps the most significant is that of the relatively poor separation efficiencies displayed for smaller analytes due to poor mass transfer characteristics. Much work has been done to elucidate the relationship between porosity, flow characteristics and mass transfer within the monolith [160,161,162,163], particularly for the separation of smaller molecules. Tracer molecules such as uracil have often been used to study the mass transport and retention characteristics of small molecules in experiments which show the dispersion of the analyte or tracer in the column with respect to elution time [162]. In addition, for the separation of large biomolecules, most separations are performed in gradient mode, improving peak sharpness due to reduced adsorption of the biomolecule. However, the use of gradient elution means that comparisons between individual monolith samples cannot be reliably performed due to the different flow rates, solvent compositions and pressures utilized between different runs. Therefore, the ideal method for comparison purposes would involve an isocratic separation, however, this may not lead to the best separation, but one which can be reliably compared to other separations using similar monolith samples.

#### 3.3.2. Chemical Compositional Variations

Another important limitation of these materials can be traced back to their preparation, since the crosslinking and polymerization can be considered to be uncontrolled processes; this leads to materials with variable degrees of crosslinker content throughout the monolith. Therefore, while the chemical functionality of the monolith can be controlled by established chemistries, as we shall see later, the overall composition of the polymer and crosslinker is much more difficult to control, and compositional variations are therefore observed [83]. This remains one of the most exciting challenges facing monolith scientists, achieving control over the exact chemical composition of the monolith.

#### 3.3.3. Physical Architecture Variations

The effect of monomer and crosslinker concentration on the phase separation and resulting physical architecture, including porosity of the monolith, are well documented [161,164]. Increased temperature generally leads to a reduction in the average pore diameter, due to increased polymer solvation, as well as lower void volumes due to an increased number of nuclei, due in turn to higher polymerization rates at increased temperature. Macropores generally allow for better fluid flow, however they are only a small percentage of the overall porosity. Mesopores contribute to the bulk of the porosity observed and may also form connecting channels between macropores. However, there are conflicting views on whether mesopores actively contribute to a more efficient separation or whether it is surface area and therefore the number of interaction sites, which is the dominant factor. It has been noted that flow near the walls can also play a role in the separation efficiency due to the degree of bonding of the monolith to the capillary walls and the potential for variations in porosity level. Therefore, while a particular morphology may appear to be well oriented and particularly uniform, the true measure of its chromatographic suitability can only be assessed from the efficiency of the resulting separation, in terms of theoretical plate count or the corresponding van Deemter plot.

The size of the polymer globules formed can affect the porosity, and therefore the flow characteristics. If this increases dispersion, then peak broadening will occur and resolution will be diminished. It can be stated that while the chemical functionalization dictates the interaction and retention of analytes on the monolith and the elution order, the physical architecture of the monolith is also hugely important as this affects the flow dynamics, dispersion, peak shape and retention. In order to understand whether any stationary phase is suitable for a desired separation application, the flow dynamics and mass transfer in the media should be well understood. The effect of the architecture on any separation is complex and multivariate in nature. Tortuosity, pore size distribution and overall porosity will affect the flow dynamics and mass transport. Tortuosity refers to the path a molecule takes through the porous structure. Smaller molecules tend to be trapped in pores more easily and take a longer time to elute than do larger molecules. The somewhat random polymerization nature of current methods of monolith fabrication means that the architecture and flow through two monoliths, produced using nominally similar conditions, will be different. This will result in differences in the corresponding flow dynamics through these. One of the main differences between polymer monolithic stationary phases is the eddy and random diffusion rates due to the high degree of tortuosity. Considering the van Deemter equation, (Equation (2)), this means that the “A” and “B” terms are always going to be high which contributes to a higher theoretical plate height.

SEM and TEM data, while useful, only show a small part of the overall monolith in most cases and therefore have limited ability to predict the physical structure of the rest of the monolith, and therefore its effect on the separation performed. However, 3D imaging techniques, such as with serial-block face SEM, have demonstrated the potential to capture the monolith physical architecture including the porosity distribution [82,165]. Such 3D digital reconstructions of monolith structures will allow the development of realistic and useful computational models for flow analysis and mass transport characteristics. This analysis will allow for correlations between fabrication parameters and resulting monolith characteristics. However, it is noted that in some cases larger pores could potentially be ignored or overlooked as being the result of damage to the sample incurred during preparation for SEM analysis, and that MIP analysis is based on the least constricted entry point of the pore, giving a somewhat skewed analysis of its true size.

## 4. Conclusions and Outlook

This review paper serves to give the reader an overview of some important developments over the last 20 years in the preparation and application of methacrylate monoliths for separation and purification of chemical and biological species. This particular branch of polymer monolith has received more research and development attention, as well as applications than other types of polymer and non-polymer based monoliths. This can be seen to be due to the advantages of methacrylate-based monoliths. They can be prepared via a number of synthetic routes, and be functionalized with a wide variety of chemical (ion exchange groups, alkyl chains, ethers, diols, OH groups), as well as biological functional groups such as proteins and other bio-affinity ligands. Therefore tailoring their chemical interactions is possible and has been achieved for a wide variety of application as noted in this review. The ease of functionalization and preparation of methacrylate monoliths has been a key factor in their appeal to researchers, however it must be stressed that this work is still very much in the early stages and significant scope remains for improvement of existing chemical modifications in terms of control and in the discovery of new chemical transformations and polymerization control mechanisms. Of more importance and which ultimately will have a greater impact on the success of methacrylate monoliths is a greater understanding of effects of the physical architecture of the monolith on the chromatographic separation or amplification of analytes. An understanding of the physical architecture would involve fully understanding the complex interplay of flow, mass transfer, tortuosity, monolith dimensions, diffusion processes, porosity and pore size distribution, as well as network homogeneity and relating all of these to the subsequent separation and to the fabrication method employed in each case. Some steps have been taken in this direction but more work needs to be done.

It can be seen that methacrylate based monolithic stationary phases have been examined in detail for both chemical and biological separations. This development has overcome some perceived shortcomings and led to valuable commercial outputs, such as CIM™, as well as the development of methods which have been validated for current Good Manufacturing Practice (cGMP) in the pharmaceutical and other industrial sectors by many research groups [140,146]. In particular, methacrylates have been shown to be adept in the separation and amplification of biological species and in some cases have significant potential for industrial applications. The possibility of scaling up production of monoliths to multi-liter volumes has also been investigated [146,166]. The suitability of methacrylates for the separation of small molecules is still a matter of debate, but some examples of separations involving alkylbenzenes, herbicides and pharmaceuticals have been reported. Innovative fabrication methods, such as e-beam curing, and 3D printing, have been utilized alongside more tried and tested polymerization reactions.

One other attractive feature of methacrylate based monolithic media comes from the fact that application areas are varied ranging from detection of herbicides, pharmaceuticals, pesticides, and acidic and basic molecules to proteins, peptides, amino acids and DNA in various forms.

A key factor in this area of improvement is a better understanding of the structure of the monoliths. Commonly used techniques such as SEM and TEM should be increasingly complemented by other sectioning and computer reconstruction of the scanned layers to produce a 3D digital topographic reconstruction of the porous structure [82,165]. However, correct interpretation of the data is crucial. This characterization technique in conjunction with advances in 3D printing could lead to reproducible production of well-defined macroporous and microporous structures. Such structures would possess well-defined tortuosity and inter-channel diffusion characteristics, based on tracer studies, SEM serial face sectioning, micro-tomology and computer reconstruction data. Using design of experiment methodology, multiple monolith physical architectures could be evaluated until optimized structures are achieved. By having a “model” monolith, displaying optimized separation efficiencies for specific species, additive-manufacturing techniques could then be employed to create multiple copies of this ideal monolith. While this research area is only in the early stages of development this research could provide new methods of monolith production. This could be one where the amount of cross-linker is well defined and homogenous throughout the monolith material and where the pore structure and distribution can be tightly controlled. Well-defined dimensional repeating structures could also be produced with controlled macropore sizes. Some two photon additive manufacturing systems can currently achieve 500 nm lateral resolution [167]. It is therefore reasonable to assume that well defined macroporous structures at the smaller end of the scale will soon be within reach. Monolith development can be seen to benefit therefore from an interdisciplinary approach. Synthetic chemists, chromatography chemists, biologists, as well as precision and systems integration engineering experts working closely together will ensure that advancements in each discipline can be harnessed in order for optimization of novel advancements to be made in the future development and application of methacrylate monoliths.

## Abbreviations

ABS	Acrylonitrile Butadiene Styrene
AHA	6-Azido Hexanoic Acid
AIBN	Azo bis-isobutyronitrile
APTES	(3-Aminopropyl)triethoxysilane
ARTP	Atom Transfer Radical Polymerization
BET	Brunauer-Emmett-Teller
BSA	Bovine Serum Albumin
BuMA	Butyl Methacrylate
CA	Carbonic Anhydrase
CAD	Computer Aided Design
CD	Cyclodextrin
CEC	Capillary Electro Chromatography
CIM	Convective Interactive Media
COC	Cyclic Olefin Co-polymer
conA	conAlbumin
CuAAC	Cu(I) catalyzed 1,3 dipolar Azide-Alkyne cycloaddition
CVD	Chemical Vapour Deposition
DEAE	di-Ethyl Amino-Ethyl
DNA	Deoxyribonucleic acid
EDMA	Ethylene DiMethacrylate
EDX	Energy Dispersive X-ray Spectroscopy
FIA	Flow Injection Analysis
FIB	Focussed Ion Beam
FITC	Fluorescein Iso-Thiocyanate
GC	Gas Chromatography
GMA	Glycidyl Methacrylate
GMP	Good Manufacturing Practice
GO	Graphene Oxide
HEPES	4-(2-Hydroxyethyl)piperazine-1-ethanesulfonic acid
HIC	Hydrophobic Interaction Chromatography
HILIC	Hydrophilic Interaction Liquid Chromatography
HIPE	High Internal Phase Emulsions
HPAA	Polyacrylic acid
HPLC	High Performance Liquid Chromatography
HRP	Horseradish Peroxidase
HSA	Human Serum Albumin
INS	Insulin
LSCM	Laser Scanning Confocal Microscopy
MALDI-TOF	Matrix Assisted Laser Desorption/Ionization—Time Of Flight
MB	Myoglobin
MIM	Molecular Imprinted Monolith
MMA	Methyl Methacrylate
OVA	Ovalbumin
PAH	Polycyclic Aromatic Hydrocarbons
PCR	Polymerase Chain Reaction
PDMS	Poly (di-Methyl Siloxane)
PEEK	Poly Ether Ether Ketone
PEG	Poly(Ethylene Glycol)
PEGDA	Poly Ethylene Glycol di-Acrylate
PLOT	Porous Layer Open Tubular Column
PMMA	Poly Methyl Methacrylate
PP2	Phosphatase B,
PTFE	Poly Tetra Fluoro-Ethylene
PVA	Poly Vinyl Alcohol
RNA	Ribonucleic acid
RT-qPCR	Real Time quantitative Polymerase Chain Reaction
RP-LC	Reverse Phase Liquid Chromatography
SEM	Scanning Electron Microscopy
SPE	Solid Phase Extraction
TLC	Thin Layer Chromatography
UTLC	Ultra Thin Layer Chromatography
